# Modeling Dynamic Recrystallization Behavior in a Novel HIPed P/M Superalloy during High-Temperature Deformation

**DOI:** 10.3390/ma15114030

**Published:** 2022-06-06

**Authors:** Qiu-Mei Yang, Yong-Cheng Lin, Ming-Song Chen, Zi-Jian Chen

**Affiliations:** 1 School of Mechanical and Electrical Engineering, Central South University, Changsha 410083, China; yangqm@csu.edu.cn (Q.-M.Y.); chenms18@csu.edu.cn (M.-S.C.); zjchen@csu.edu.cn (Z.-J.C.); 2 State Key Laboratory of High-Performance Complex Manufacturing, Changsha 410083, China

**Keywords:** P/M superalloy, microstructure evolution, grain growth, dynamic recrystallization

## Abstract

The dynamic recrystallization (DRX) features and the evolution of the microstructure of a new hot isostatic pressed (HIPed) powder metallurgy (P/M) superalloy are investigated by hot-compression tests. The sensitivity of grain dimension and DRX behavior to deformation parameters is analyzed. The results reveal that the DRX features and grain-growth behavior are significantly affected by deformation conditions. The DRX process is promoted with a raised temperature/true strain or a reduced strain rate. However, the grains grow up rapidly at relatively high temperatures. At strain rates of o.1 s^−1^ and 1 s^−1^, a uniform microstructure and small grains are obtained. Due to the obvious differences in the DRX rate at various temperatures, the piecewise DRX kinetics equations are proposed to predict the DRX behavior. At the same time, a mathematical model for predicting the grain dimension and the grain growth behavior is established. To further analyze the DRX behavior and the changes in grain dimension, the hot deformation process is simulated. The developed grain-growth equation as well as the piecewise DRX kinetics equations are integrated into DEFORM software. The simulated DRX features are consistent with the test results, indicating that the proposed DRX kinetics equations and the established grain-growth model can be well used for describing the microstructure evolution. So, they are very useful for the practical hot forming of P/M superalloy parts.

## 1. Introduction

Owing to their excellent anti-corrosion/fatigue properties and strengths, powder metallurgy (P/M) superalloys are widely applied for manufacturing the turbine disks of aero-engines [1,2]. However, due to the high content of alloying elements and the internal defects in P/M superalloys, hot forming processes, for example, hot-extrusion and forging processes, are fairly complex [3]. Furthermore, the hot-forming process is usually accompanied by complicated deformation mechanisms such as dynamic recrystallization (DRX), dynamic recovery (DRV), etc. These deformation mechanisms are greatly influenced by the forming parameters [4,5,6]. Therefore, to precisely tailor the microstructures and optimize the final properties of alloy parts, it is necessary to research the sensitivity of microstructures and properties to deformation parameters. Also, accurate models should be established to predict the microstructures in hot deformed alloys [7].

During hot deformation, the microstructures can be refined and the metallurgical defects can be efficiently removed. Up until now, the material flow characteristics [8,9] and microstructure evolution [10,11] of nickel-base superalloys have been widely reported. Fang et al. [12] studied the two-pass rheological characteristics and DRX behavior of the hot-extruded P/M alloy. Zhang et al. [13] revealed the interaction between the flow behavior and γ′ phase of the FGH96 superalloy and optimized the hot forming parameters. Wang et al. [14] investigated the recrystallization and densification behavior of a Ni-based superalloy in the forging process. Zhang et al. [15] discussed the role of non-uniform precipitates on local plastic deformation in hot isostatic-pressed (HIPed) FGH96 alloy. Sreenu [16] investigated the microscopic structure features in a new P/M superalloy which was processed by the HIP route. In addition, the mathematical model is a vital way to depict the relationship between deformation conditions and rheological features, as well as the microscopic structure evolution in alloys [7,17,18,19,20]. Meanwhile, accurate mathematical models are essential to analyze/forecast material deformation behavior and the evolution of the microstructure by the finite element method (FEM) [21,22,23]. Recently, for the deformed P/M superalloys, some accurate mathematical models were established. For example, Liu et al. [24,25] established an equation to depict the interaction between the forming conditions and the DRX grain dimension of a hot-deformed FGH96 superalloy. Zhang et al. [26] developed the strain-compensated constitutive and ANN models to forecast the rheological characteristics for a hot-extruded P/M superalloy. Tan et al. [27] revealed that the DRX nucleation behavior and the evolution of the microstructure are sensitive to the Zener–Hollomon (Z) parameter in a hot-extruded P/M superalloy.

Though the deformation mechanisms as well as the microstructure evolution of HIPed P/M superalloys have been reported, accurate models for predicting the DRX features and grain-growth behavior are still rarely reported. In this work, the hot-compression experiments of a novel HIPed P/M superalloy are conducted. Then, the flow features, DRX mechanism, and grain features of an advanced P/M superalloy are systematically investigated. The grain dimension and DRX volume fraction (*X*_drx_) are quantitatively calculated. The piecewise DRX kinetics equations and grain-growth model are established to predict the DRX and grain-growth behaviors under different deformation conditions. Then, the developed models are integrated into DEFORM software. Finally, the DRX features and microstructure evolution during hot compression are simulated.

## 2. Material and Experiment Procedures

**Figure 1:**
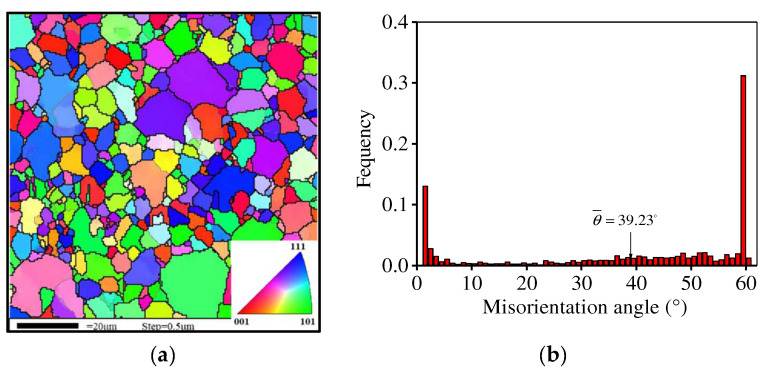
Initial microstructure of the HIPed superalloy: (**a**) EBSD-OIM map; (**b**) Misorientation angle distributions.

The elemental components (wt. %) for the novel P/M superalloy are listed in Table 1. The size of the initial powder is about 80 μm. The test material was produced through argon atomization and hot isostatic pressing at a temperature of 1150 °C and a pressure of 150 MPa for 4 h. Figure 1 displays the primary microstructure of the HIPed P/M superalloy. The equiaxed and coarse grains are observed and the mean grain dimension is about 9.5 μm. The deformed cylinder samples were obtained from the HIPed superalloy and their radius was 4 mm and their height was 12 mm. Isothermal compression experiments were executed on the Gleeble-3500D simulator. The range of deformation amount was from 20% to 60% and that of the strain rate was from 1 to 0.001 s^−1^. The temperature was selected from 1080 to 1170 °C. Additionally, the graphite slices were employed to minimize the friction between the molds and sample.

**Figure 2:**
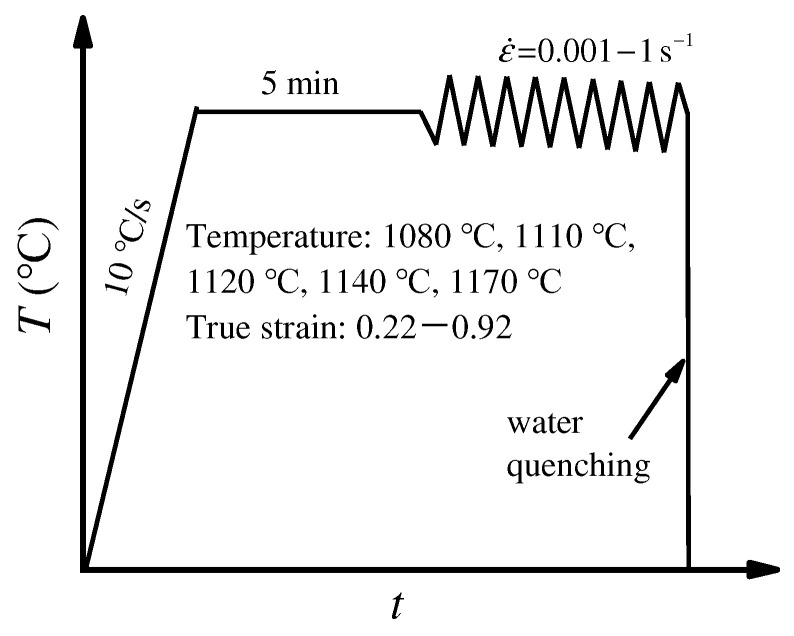
Schematic diagram of hot compression tests for the HIPed superalloy.

The schematic plot of hot-deformation experiments is illustrated in Figure 2. To investigate the DRX behavior and the grain dimension in the hot-compression process, electron back-scattered diffraction (EBSD, JEOL-7001F1 FE-SEM) was applied to observe the deformed microstructures. The method of preparing EBSD samples has been reported in detail in our previous study, i.e., the samples were mechanically polished and then electrolytically polished by a solution of 10 mL HClO_4_ and 90 mL CH_3_CH_2_OH at a temperature of −31 to −26 °C as well as a voltage of 23 V [28]. The EBSD test was performed at an acceleration voltage of 25 kV, step size of 0.5 μm, and a scanning area of 100 μm × 100 μm. The HKL Channel 5 software was applied to deal with EBSD data. The *X*_drx_ and grain size were accurately calculated by MTEX5.7.0.

## 3. Results and Discussion

### 3.1. Rheological Characteristics and Deformation Mechanisms

**Figure 3:**
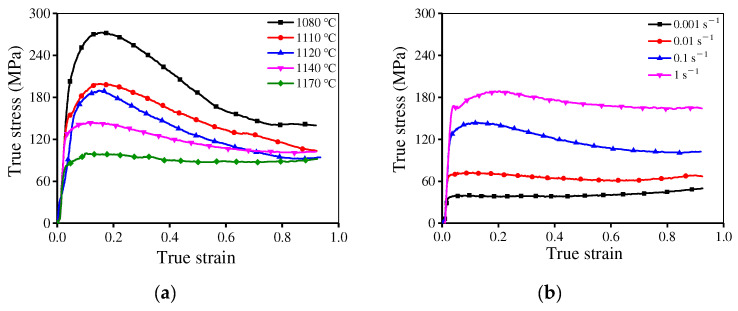
Typical true stress–strain curves of the HIPed superalloy at (**a**) $\dot{\varepsilon }=0.1\;s^{-1}$; (**b**) T = 1140 °C.

Figure 3 illustrates the rheological stress of the researched superalloy under the tested conditions. The stress is large at low temperatures or high strain rates. This is because the grain boundaries (GBs) migration is weakened at low temperatures and the deformation time is short at high strain rates. In addition, the work-hardening (WH) behavior becomes obvious with a raised strain rate or a reduced temperature [29,30]. In the early period of deformation, the stress increases instantly due to the WH induced by rapid dislocation proliferation and accumulation [31,32]. As the true strain is raised, the dynamic recovery (DRV) and DRX become obvious, which results in decreased stress. As the deformation continues, stable stresses are obtained because of the kinetic equilibrium between WH, DRX, and DRV [33]. Particularly, the rheological stress exhibits a fast drop at 1120 °C/0.1 s^−1^. This is caused by heterogeneous deformation or local deformation heating or cracking [34,35].

### 3.2. The Evolution of Microstructure

#### 3.2.1. Influence of True Strain on DRX Behavior and Grain Dimension

The grain-orientation spread (GOS) method can reflect the orientation gradient within grains and evaluate the DRX degree in the deformed alloy [36]. Here, the DRX and deformed grains are recognized by the GOS method [37]. The calculation equation is expressed as [38]:

(1) $$\mathrm{GOS}=\frac{1}{J\left(a\right)}\sum_{b}\omega_{ab}$$

where J(a) shows the pixels amount in grain *a* and $\omega_{ab}$ shows the misorientation degree between the orientation of pixel position *b* and average orientation of grain *a*. According to the GOS distribution of the complete DRX sample and GOS division principle [39], the grains with ${\mathrm{GOS}\;<\;3}^{\circ }$ are defined as DRX grains.

**Figure 4:**
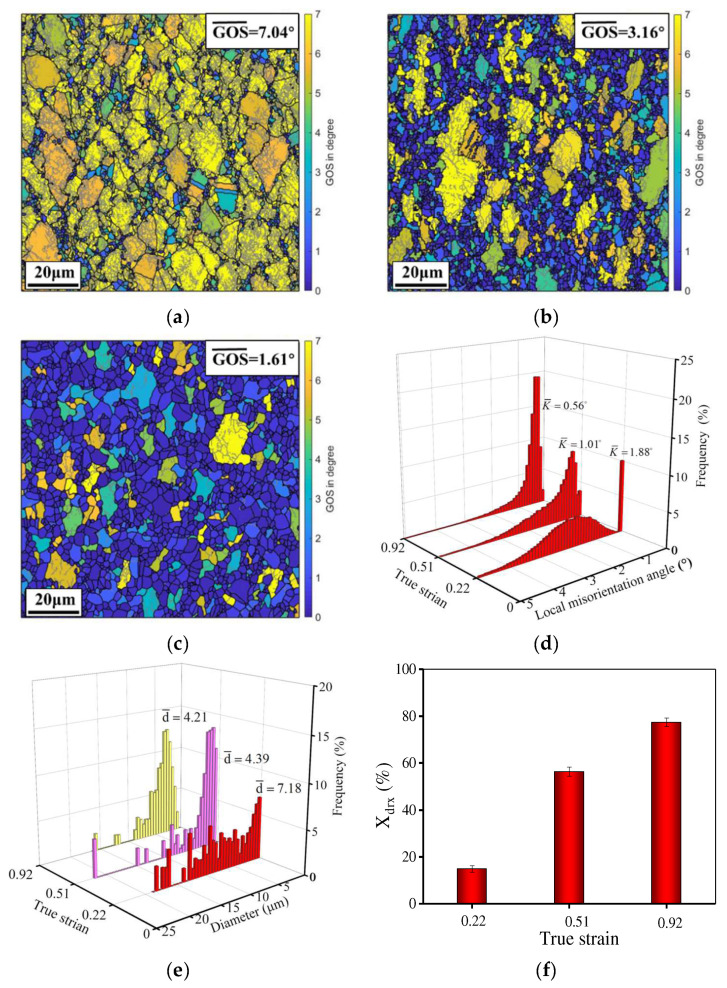
GOS maps of the HIPed superalloy deformed at the true strain of (**a**) 0.22; (**b**) 0.51; (**c**) 0.92 [28]; (**d**) the local misorientation angle; (**e**) average grain size distribution; (**f**) area fraction of DRX (HAGBs and LAGBs correspond to the black line and gray line, respectively. The temperature and strain rate are 1110 °C and 0.1 s^−1^, respectively).

When the temperature and strain rate are 1110 °C and 0.1 s^−1^, respectively, the GOS distribution at the true strain of 0.22, 0.51, and 0.92 is demonstrated in Figure 4. Obviously, there are some changes in DRX behavior and grain dimension at different strains. As the true strain is raised, the mean GOS and average kernel misorientation (KAM) decreases but the *X*_drx_ increases. Meanwhile, the average grain dimension is reduced from 7.18 to 4.21 μm when the strain increases from 0.22 to 0.92, whereas the mean DRX grain dimension (*d*_drx_) increases. At 0.22 (Figure 4a), a mass of substructures and serrated GBs appear. Meanwhile, fine DRX grains and necklace structures are observed at the serrated/bulging GBs because the serrated GBs have a high local orientation or strain gradient for DRX nucleation. Obviously, discontinuous dynamic recrystallization (DDRX) occurs [40,41]. At 0.22, the GOS (7.04°)/KAM (1.88°) values are relatively high and the *X*_drx_ (15.56%) is low. However, the average grain size is large, which is attributable to the high dislocation density and the low-deformation storage energy. The original GBs are gradually covered by DRX grains and the mean GOS and KAM rapidly decrease when the true strain is 0.51. In addition, the *X*_drx_ increases significantly. Although the DRX grains grow up, the average grain size decreases to 4.39 μm because of the annihilation/rearrangement of dislocations and the migration of GBs [42,43,44]. When the true strain is further raised to 0.92 (Figure 4c), the number of grains with high GOS values further decreases and the KAM also declines to 0.56°. In addition, the DRX degree increases. As the DRX grains further grow up, a uniform microstructure is obtained.

#### 3.2.2. Influence of Strain Rate on the DRX Behavior and Grain Dimension

**Figure 5:**
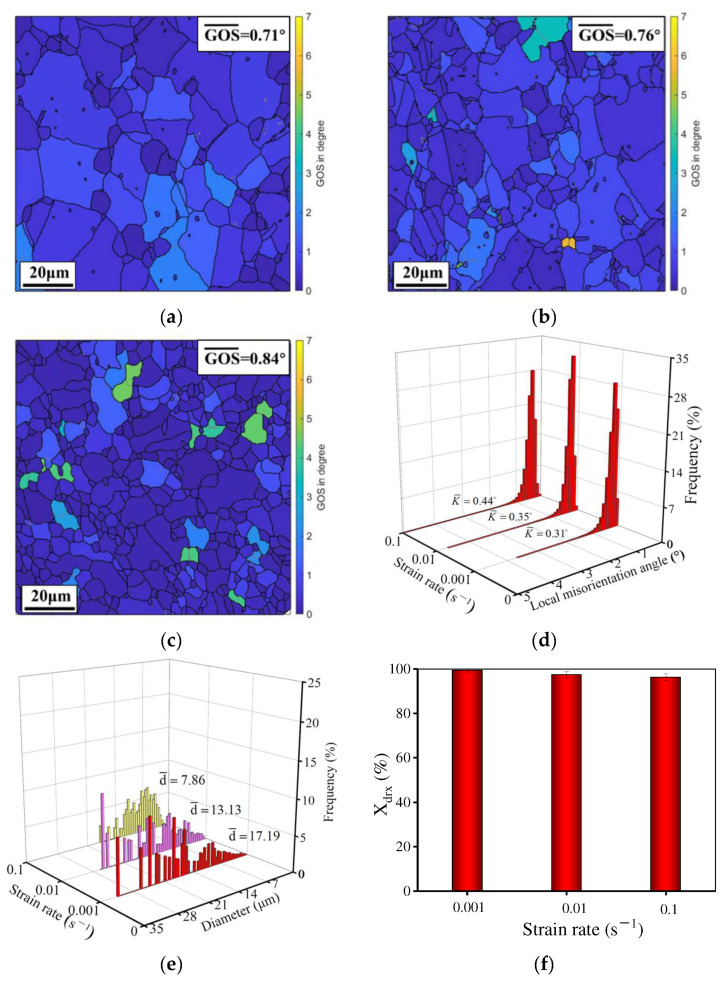
GOS maps of the HIPed superalloy deformed at a strain rate of (**a**) 0.001 s^−1^; (**b**) 0.01 s^−1^; (**c**) 0.1 s^−1^ [28]; (**d**) local misorientation angle; (**e**) average grain size distribution; (**f**) area fraction of DRX (the temperature and true strain are 1140 °C and 0.92, respectively).

Figure 5 displays the evolution of DRX behavior and grain dimension at diverse strain rates. Here, the true strain is 0.92 and the temperature is 1140 °C. Some serrated GBs and tiny DRX grains can be found, which reveals the occurrence of DDRX [34]. As the strain rate increases from 0.001 s^−1^ to 0.1 s^−1^, the average KAM and GOS increase but the *X*_drx_ decreases. Meanwhile, tiny DRX grains are found and the mean grain dimension decreases because the large strain rate can produce high strain energy and accelerate the development of substructures with a mass of dislocations. It is conducive to DRX nucleation [45]. In addition, previous reports show that less time for deformation and γ′ phase pinning results in a low *X*_drx_ and fine grains at a high strain rate [46]. The grains with high GOS have hardly been observed at 1140 °C and the *X*_drx_ at three strain rates are higher than 95%, which indicates the DRX is complete.

#### 3.2.3. Influence of Temperature on the DRX Behavior and Grain Dimension

**Figure 6:**
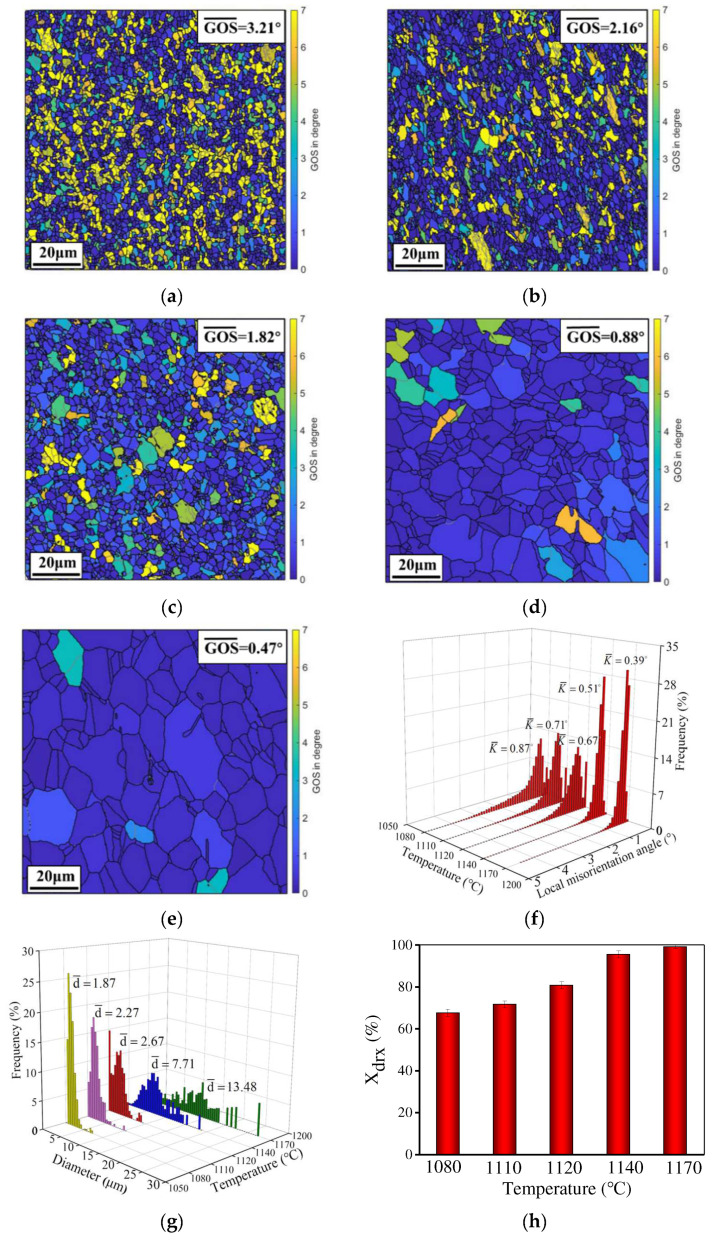
GOS maps of the HIPed superalloy deformed at temperatures of (**a**) 1080 °C; (**b**) 1110 °C [28]; (**c**) 1120 °C; (**d**) 1140 °C; (**e**) 1170 °C; (**f**) the local misorientation angle; (**g**) average grain size distribution; (**h**) area fraction of DRX (the strain rate is 1 s^−1^ and true strain is 0.92).

When the true strain is 0.92 and the strain rate is 0.1 s^−1^, the relationship between temperature and GOS is depicted in Figure 6. The mean value of GOS declines but the average grain size and *X*_drx_ increase when the temperature is raised. At low temperatures (Figure 6a,b), the grains with high GOS and fine DRX grains are observed and the KAM is high (0.87° and 0.71°). It indicates the high dislocation density in grains and the limited DRX. When the temperature is raised to 1140/1170 °C (Figure 6c,d), the enhanced DRX induces a decrease in the mean GOS and KAM. Also, the *X*_drx_ increases significantly (Figure 6f). This is attributed to the high temperature enhancing the movement of GBs and the mobility/diffusion of dislocations. In addition, the mean grain size increases to 13.48 μm due to the rapid growth of DRX grains. The γ′ phase is sufficiently dissolved, which weakens the pinning effect on GBs [47]. Particularly, the DRX rate is significantly disparate at diverse temperatures. In Figure 6h, when the temperature is below 1120 °C, the *X*_drx_ is lower than 82%. However, the DRX is almost complete at 1140 °C.

**Figure 7:**
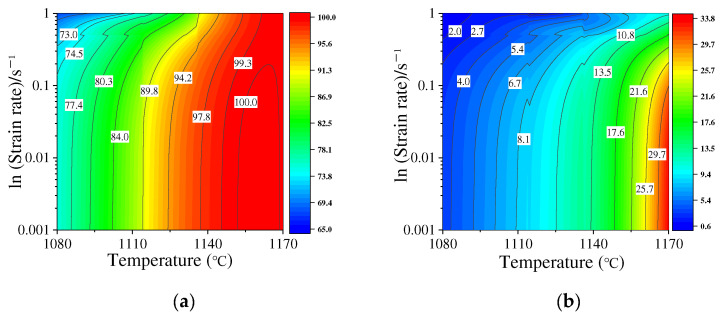
Contour maps at different deformation conditions: (**a**) DRX volume fraction; (**b**) average DRX grain size.

Summarily, the DRX behavior and grain features of the studied HIPed P/M superalloy are dramatically affected by the deformation amount, temperature, and strain rate. Hence, the contour maps to depict the effects of deformation conditions on DRX volume fraction and average DRX grain size are demonstrated in Figure 7. Obviously, the DRX is enhanced with the raised temperature or the reduced strain rate. The colors and values of the contour maps are very different in terms of the raised temperature when the strain rate is constant. When the temperature is below 1120 °C, a weak DRX is observed. However, a full DRX can be achieved at 1140/1170 °C. Also, the DRX rates are different at various temperatures. This is because the movement of GBs is enhanced by the time and energy at high temperatures. Therefore, based on the effects of temperature on DRX mechanisms, the piecewise DRX kinetics models are proposed in Section 3.3.

### 3.3. DRX Kinetics Model

Generally, the critical strain ($\varepsilon_{c}$) corresponding to DRX is decided by θ−σ curves [34,48]. Here, $\theta =\frac{d\sigma }{d\varepsilon }$ shows the WH rate, σ represents the true stress, and ε is the true strain. According to Poliak’s study [38], the $\varepsilon_{c}$ for DRX is equal to the minimum spot on the θ−σ curve and *θ* is expressed as a third-order polynomial function:

(2) $$\theta {=A}_{1}\sigma^{3}+A_{2}\sigma^{2}+A_{3}\sigma {+A}_{4}$$

where A_1_, A_2_, A_3_, and A_4_ represent material constants.

Then, Equation (2) is also expressed as:

(3) $$\frac{d^{2}\theta }{d\sigma^{2}}={6A}_{1}\sigma +{2A}_{2}$$

For $\frac{d^{2}\theta }{d\sigma^{2}}=0$, the critical stress ($\sigma_{c}$) can be expressed as:

(4) $$\sigma_{c}=-A_{2}/3A_{1}$$

**Figure 8:**
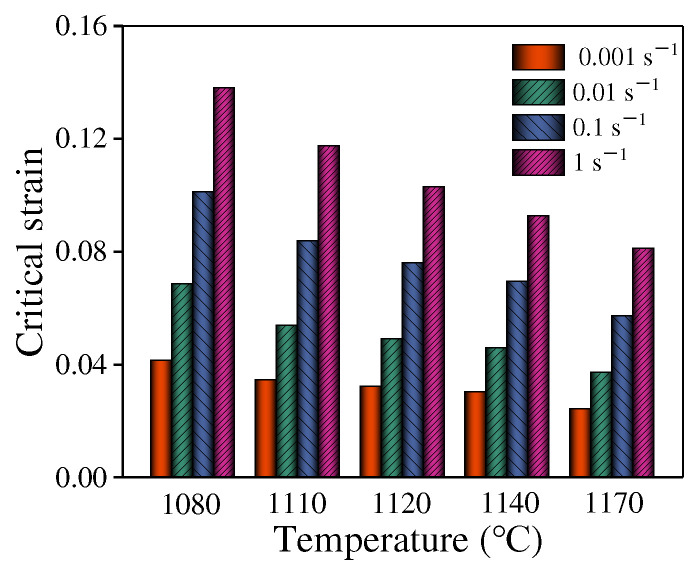
The column bars of $\varepsilon_{c}$ at different temperatures and strain rates.

Based on the measured rheological stresses, the $\sigma_{c}$ and $\varepsilon_{c}$ can be determined. Figure 8 displays the values of under various conditions. Obviously, the reduced temperature or the raised strain rate increase $\varepsilon_{c}$.

**Figure 9:**
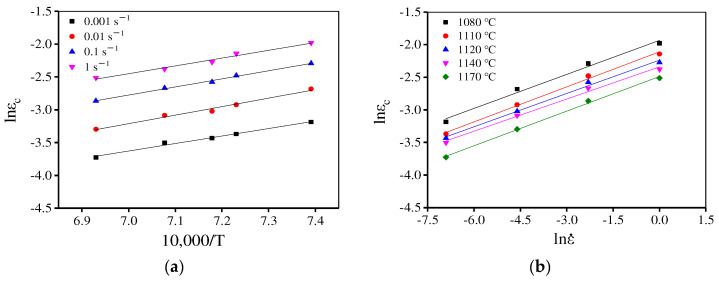
The relationship between (**a**) $\ln\varepsilon_{c}$–10,000/T and (**b**) $\ln\varepsilon_{0.5}-\ln\dot{\varepsilon }$.

Generally, $\varepsilon_{c}$ can be evaluated by:

(5) $$\varepsilon_{c}=a_{1}{\dot{\varepsilon }}^{l_{1}}\exp\;(\frac{Q_{1}}{RT})$$

where R is the constant for gas (8.314 J/(K·mol)). In addition, $a_{1}$ and $Q_{1}$ are material parameters, which can be decided by least square linear fitting of $\ln\varepsilon_{c}$–10,000/T and $\ln\varepsilon_{c}$ − $\ln\dot{\varepsilon }$ plots, respectively, as displayed in Figure 9. Thus, $\varepsilon_{c}$ is determined as:

(6) $$\varepsilon_{c}=1.794\times 10^{-5}{\dot{\varepsilon }}^{0.1731}\exp\;(\frac{101,090.13}{RT})$$

**Figure 10:**
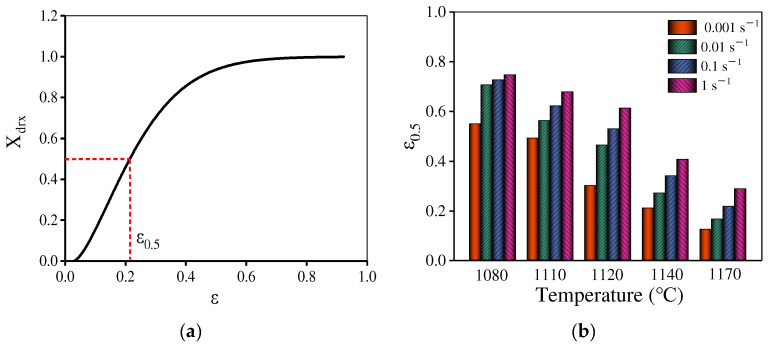
(**a**) Relationship between *X*_drx_ and ε at 1140 °C/0.001 s^−1^. (**b**) $\varepsilon_{0.5}$ at different deformation conditions.

**Figure 11:**
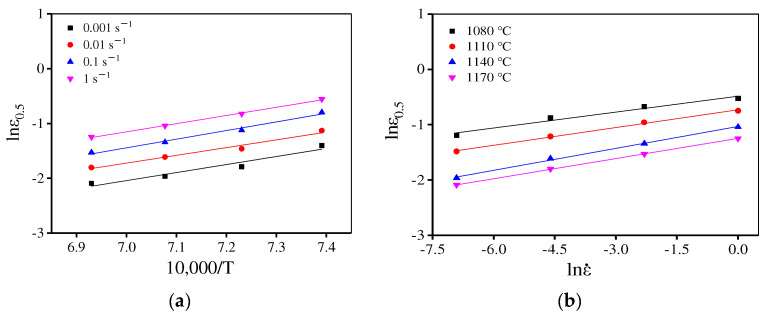
Relationship between $\varepsilon_{0.5}$ and deformation parameters (**a**) $\ln\varepsilon_{0.5}$ –10,000/T and (**b**) $\ln\varepsilon_{0.5}-\ln\dot{\varepsilon }$.

Figure 10 displays the variations of *X*_drx_ with the true strain at 1140 °C/0.001 s^−1^. The value of *X*_drx_ is small in the initial incubation stage. After this incubation period, the DRX is accelerated and finally tends to be stable. The $\varepsilon_{0.5}$ can be obtained through the $X_{\mathrm{drx}}-\varepsilon$ curve (Figure 10a). Figure 10b illustrates the value of $\varepsilon_{0.5}$ at various compression conditions. The reduced strain rate or the raised temperature can decrease $\varepsilon_{0.5}$. Similarly, $\varepsilon_{0.5}$ is related to deformation parameters [49], i.e.,

(7) $$\varepsilon_{0.5}=a_{2}{\dot{\varepsilon }}^{l_{2}}\exp\;(\frac{Q_{2}}{RT})$$

where $a_{2}$, $l_{2}$, and $Q_{2}$ represent material parameters, which can be evaluated by $\ln\varepsilon_{0.5}$–10,000/T and $\ln\varepsilon_{0.5}$ − $\ln\dot{\varepsilon }$ plots, respectively, as illustrated in Figure 11. Hence, $\varepsilon_{0.5}$ is determined as:

(8) $$\varepsilon_{0.5}=4.577\times 10^{-7}{\dot{\varepsilon }}^{0.114}\exp\;(\frac{159,420.7}{RT})$$

**Figure 12:**
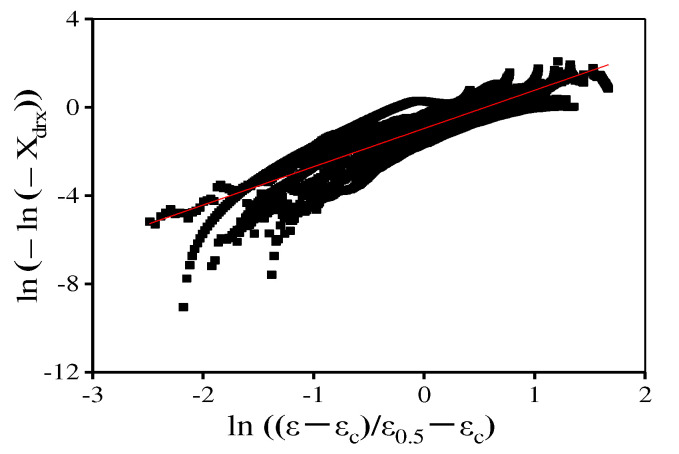
The relationship between $\ln\left(-\ln\left(1-X_{\mathrm{drx}}\right)\right)$ and $\;\ln\left(\left(\varepsilon -\varepsilon_{c}\right)/\left(\varepsilon_{0.5}-\varepsilon_{c}\right)\right)$.

In Figure 10, the variations in *X*_drx_ with the strain are similar to a sigmoidal curve, which can be described as [32]:

(9) $$X_{\mathrm{drx}}=1-\exp\;[-0.693{\left(\frac{\varepsilon -\varepsilon_{c}}{\varepsilon_{0.5}-\varepsilon_{c}}\right)}^{n}]\;\;\;\;\;\;\;\;(\varepsilon >\varepsilon_{c})$$

where n is a material constant. *X*_drx_, ε, $\varepsilon_{c}$, and $\varepsilon_{0.5}$ separately represent the DRX volume fraction, true strain, critical strain, and the strain where *X*_drx_ reaches 50%. Figure 12 displays $\ln\left(-\ln\left(1-X_{\mathrm{drx}}\right)\right)$ − $\ln\left(\left(\varepsilon -\varepsilon_{c}\right)/\left(\varepsilon_{0.5}-\varepsilon_{c}\right)\right)$ plot, and the n is determined through the linear fitting of this plot. Then, the DRX kinetics models are determined as:

(10) $$\left\{ \begin{array}{l} X_{\mathrm{drx}}=1-\exp\;[-0.693{\left(\frac{\varepsilon -\varepsilon_{c}}{\varepsilon_{0.5}-\varepsilon_{c}}\right)}^{2.15}]\\ \varepsilon_{c}=1.747\times 10^{-5}{\dot{\varepsilon }}^{0.1737}\exp\;(\frac{101,502.36}{RT})\\ \varepsilon_{0.5}=4.577\times 10^{-7}{\dot{\varepsilon }}^{0.114}\exp\;(\frac{159,420.7}{RT}) \end{array}\right.$$

**Figure 13:**
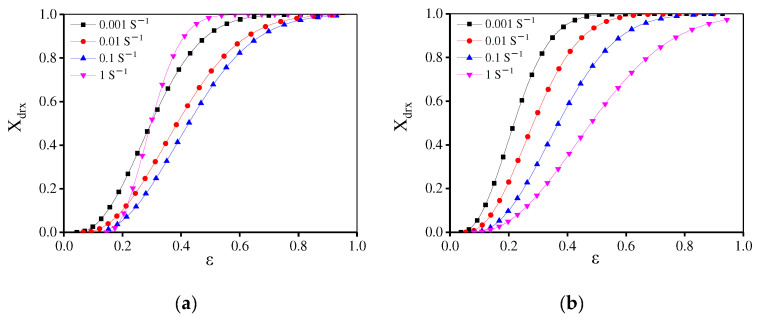

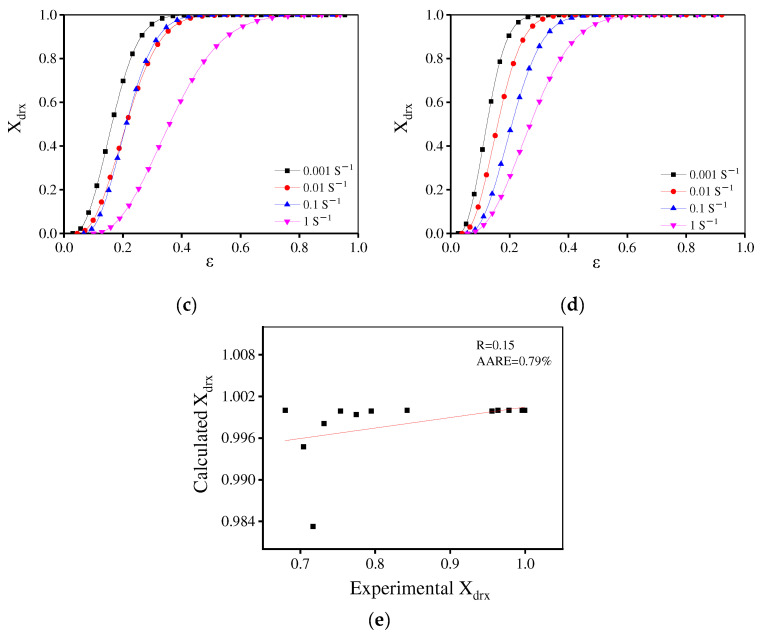
The effects of strain rate and strain on *X*_drx_ at (**a**) 1080 °C; (**b**) 1110 °C; (**c**) 1140 °C; (**d**) 1170 °C. (**e**) The comparisons between the calculated and experimental *X*_drx_.

Figure 13 gives the relationship between the strain rate/ε and the calculated *X*_drx_. Also, the comparisons between the calculated and experimental *X*_drx_ are represented. To validate the precision of traditional DRX kinetics equations, the average absolute relative error (AARE) and correlation coefficient € are calculated, i.e.,

(11) $$R=\frac{\sum_{i=1}^{N}\left(E_{i}-\bar{E}\right)\left(P_{i}-\bar{P}\right)}{\sqrt{\sum_{i=1}^{N}{\left(E_{i}-\bar{E}\right)}^{2}{\left(P_{i}-\bar{P}\right)}^{2}}}$$

(12) $$AARE=\frac{1}{N}\sum_{i=1}^{N}\left|\frac{E_{i}-P_{i}}{E_{i}}\right|\times 100$$

where *E_i_* is the measured value and *P_i_* shows the calculated one. Meanwhile, the measured mean value is expressed as *E* and the calculated mean value is defined as *P*. In Figure 13, the traditional DRX equation can well describe the DRX behavior at high temperatures (1140–1170 °C). However, when the forming temperatures are below 1110 °C, the predicted *X*_drx_ is much higher than the experimental value and the R between experimental and predicted *X*_drx_ is only 0.15. Meanwhile, the predicted $\varepsilon_{0.5}$ value is low, which indicates that DRX occurs in advance. According to the above EBSD observations, due to a large number of dissolved γ′ phases [50] and enough energy for GBs’ migration at high temperatures, DRX occurs rapidly. However, the γ′ phase is difficult to dissolve and the deformation time is short with the raised strain rate and the reduced temperature, resulting in the decreased DRX rate. Thus, the DRX rates are quite different at various deformation temperatures. So, the traditional kinetics model cannot accurately predict the DRX behavior under different deformation conditions.

**Figure 14:**
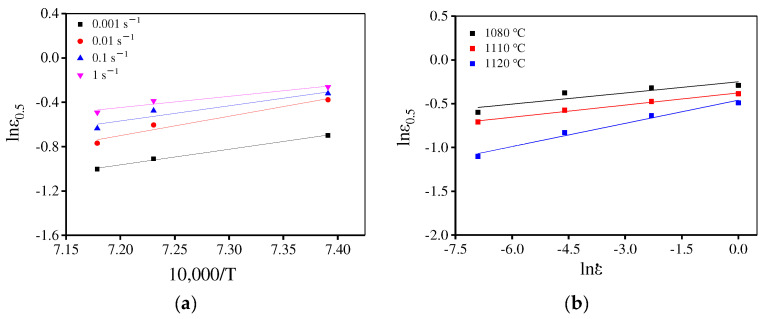
Relationship between $\varepsilon_{0.5}$ and deformation conditions (**a**) $\ln\varepsilon_{0.5}$ –10,000/T and (**b**) $\ln\varepsilon_{0.5}-\ln\dot{\varepsilon }$.

**Figure 15:**
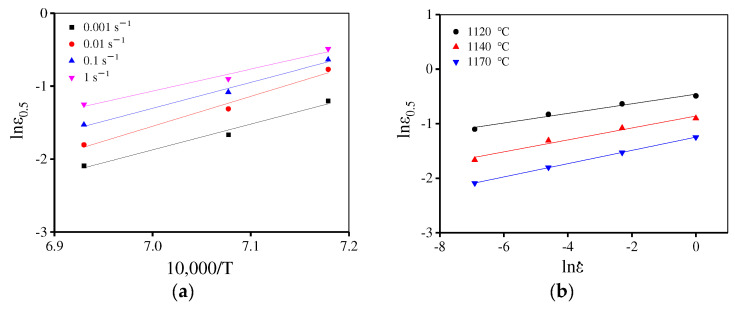
Relationship between $\varepsilon_{0.5}$ and deformation parameters (**a**) $\ln\varepsilon_{0.5}$ –10,000/T and (**b**) $\ln\varepsilon_{0.5}-\ln\dot{\varepsilon }$.

In Figure 10b, when the temperature is below 1120 °C $\varepsilon_{0.5}$ is high. However, at 1140 °C and 1170 °C, $\varepsilon_{0.5}$ is low indicating that the DRX rate is significantly different at various temperatures. Combined with the microstructure evolution discussed in Section 3.2.3, to precisely describe the DRX behavior for the present alloy, the piecewise equations can be used to predict $\varepsilon_{0.5}$ and the segmented temperature is 1200 °C. The material parameters are determined by least square linear fitting of $\ln\varepsilon_{0.5}$–10,000/T and $\ln\varepsilon_{0.5}$ − $\ln\dot{\varepsilon }$ plots, as shown in Figure 14 and Figure 15. Then, $\varepsilon_{0.5}$ is determined as:

(13) $$\varepsilon_{0.5}=3.05\times 10^{-5}{\dot{\varepsilon }}^{0.059}\exp(\frac{114,876.8}{RT})\;\;\;\;\;\;\;\;\;\;(T=1080-1120\;℃)$$

(14) $$\varepsilon_{0.5}=5.67\times 10^{-12}{\dot{\varepsilon }}^{0.107}\exp(\frac{294,773.5}{RT})\;\;\;\;\;\;\;\;\;\;(T=1120-1170\;℃)$$

**Figure 16:**
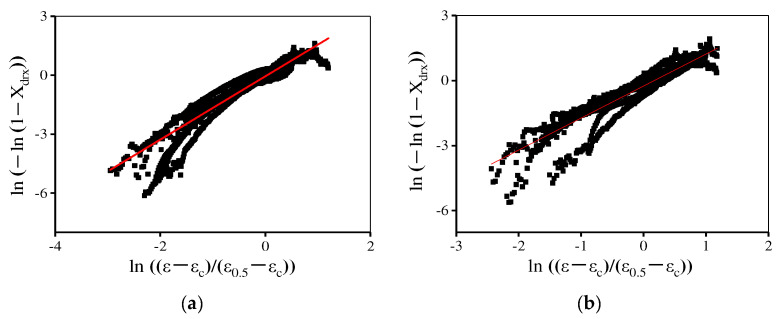
The relationship between $\ln\left(-\ln\left(1-X_{\mathrm{drx}}\right)\right)$ and $\;\ln\left(\left(\varepsilon -\varepsilon_{c}\right)/\left(\varepsilon_{0.5}-\varepsilon_{c}\right)\right)$ at (**a**) 1080–1120 °C and (**b**) 1120–1170 °C.

In Figure 16, the values of material constant n are determined in the range of 1080–1120 °C and 1120–1170 °C, respectively. Consequently, the proposed piecewise DRX kinetics equations are determined as:

(15) $$\left\{ \begin{array}{l} X_{\mathrm{drx}}=1-\exp[-0.693{\left(\frac{\varepsilon -\varepsilon_{c}}{\varepsilon_{0.5}-\varepsilon_{c}}\right)}^{n}]\\ n=\left\{ \begin{array}{l} 1.61\;\;\;\;\;\;\;\;\;(T=1080-1120\;℃)\\ 1.48\;\;\;\;\;\;\;\;(T=1120-1170\;℃) \end{array}\right.\\ \varepsilon_{c}=1.79\times 10^{-5}{\dot{\varepsilon }}^{0.173}\exp(\frac{101,090.1}{RT})\\ \varepsilon_{0.5}=\left\{ \begin{array}{l} 3.05\times 10^{-5}{\dot{\varepsilon }}^{0.059}\exp(\frac{114,876.8}{RT})\;\;\;\;\;\;\;\;\;\;\;(T=1080-1120\;℃)\\ 5.67\times 10^{-12}{\dot{\varepsilon }}^{0.107}\exp(\frac{294,773.5}{RT})\;\;\;\;\;\;\;\;\;\;(T=1120-1170\;℃) \end{array}\right. \end{array}\right.$$

**Figure 17:**
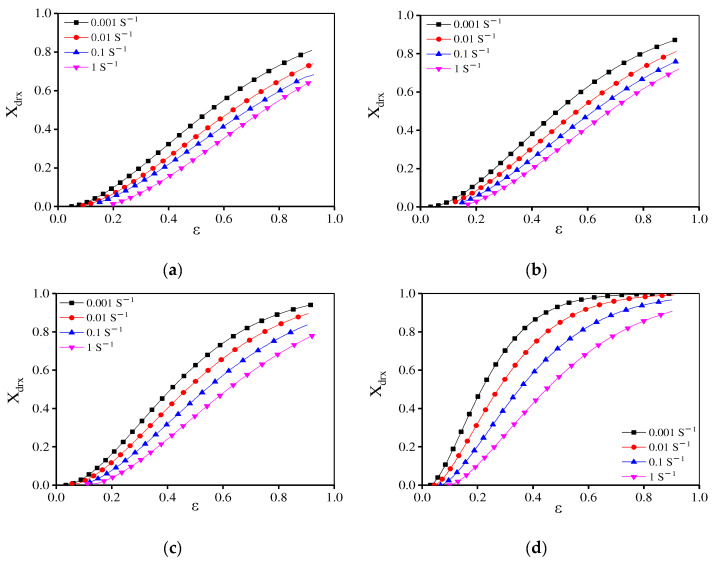

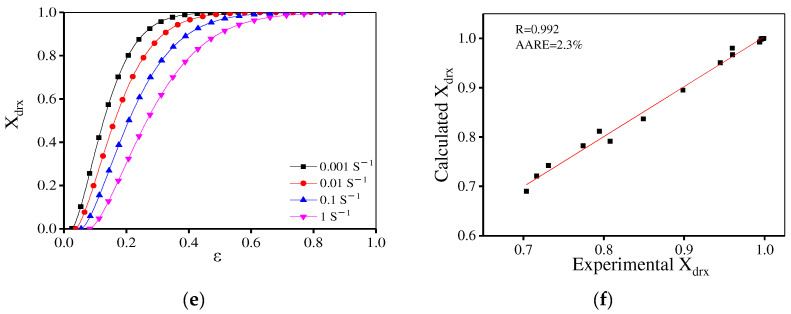
The effects of strain rate and strain on *X*_drx_ at (**a**) 1080 °C; (**b**) 1110 °C; (**c**) 1120 °C; (**d**) 1140 °C; and (**e**) 1170 °C. (**f**) The comparisons between the calculated and experimental *X*_drx_.

Figure 17 gives the variations in the calculated *X*_drx_ with the strain. Also, the comparisons between the calculated and experimental *X*_drx_ are represented. The calculated values are very close to the experimental ones. To confirm the precision of the proposed piecewise DRX kinetics equations, R and AARE are calculated. The AARE value is 2.3% and the R value is 0.992. Hence, the DRX behavior of the present HIPed P/M superalloy during hot compression can be accurately described by the proposed piecewise DRX kinetics equations.

The DRX grain size (*d*_drx_) in the stable deformation stage is listed in Table 2. Obviously, the reduced strain rate or the raised temperature can increase *d*_drx_. Generally, *d*_drx_ is connected with the compression parameters, which is evaluated through Sellars’s empirical equation [32,51]. Hence, according to experimental data, *d*_drx_ is determined as:

(16) $$d_{\mathrm{drx}}=1.76\times 10^{15}{\dot{\varepsilon }}^{-0.16}\exp\;(\frac{-393,644.47}{RT})$$

**Figure 18:**
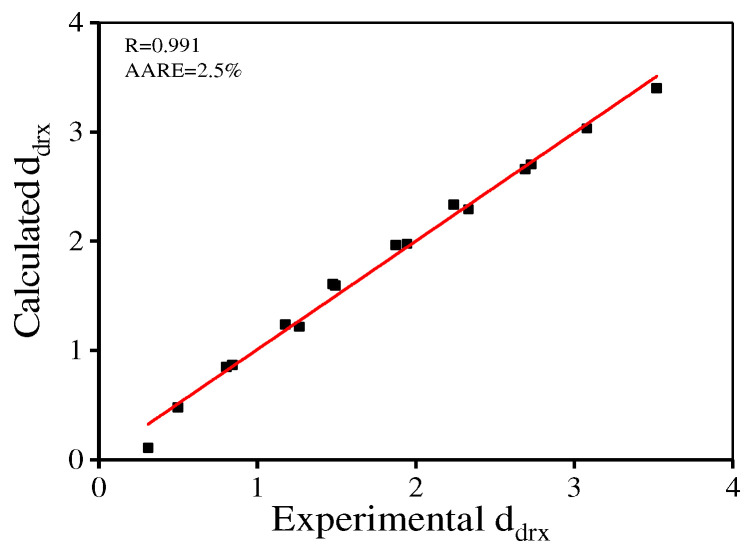
Comparisons between the calculated and experimental *d*_drx_.

Figure 18 demonstrates the comparisons between the calculated/experimental *d*_drx_. The calculated AARE value is 2.5% and the R is 0.991. Meanwhile, the mean grain dimension is demonstrated as:

(17) $$d_{i}=d_{\mathrm{drx}}X+d_{0}(1-X)$$

where $d_{\mathrm{drx}}$ represents the size of DRX grain, $d_{0}$ shows the initial grain size, and *X* represents the DRX volume fraction. Similarly, $d_{\mathrm{drx}}$ is demonstrated as:

(18) $$d_{\mathrm{drx}}=1.76\times 10^{15}{\dot{\varepsilon }}^{-0.16}\exp\;(\frac{-393,644.47}{RT})$$

### 3.4. Finite-Element Simulation of DRX Behavior and Grain-Dimension Evolution

**Figure 19:**
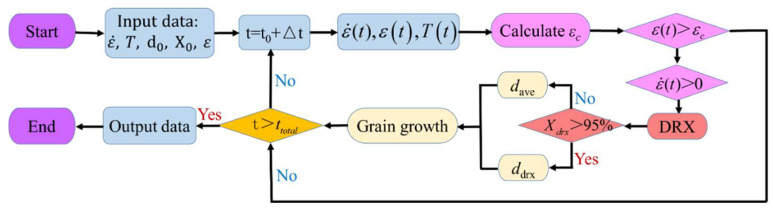
Flow chart to simulate the DRX behavior and grain size.

**Figure 20:**
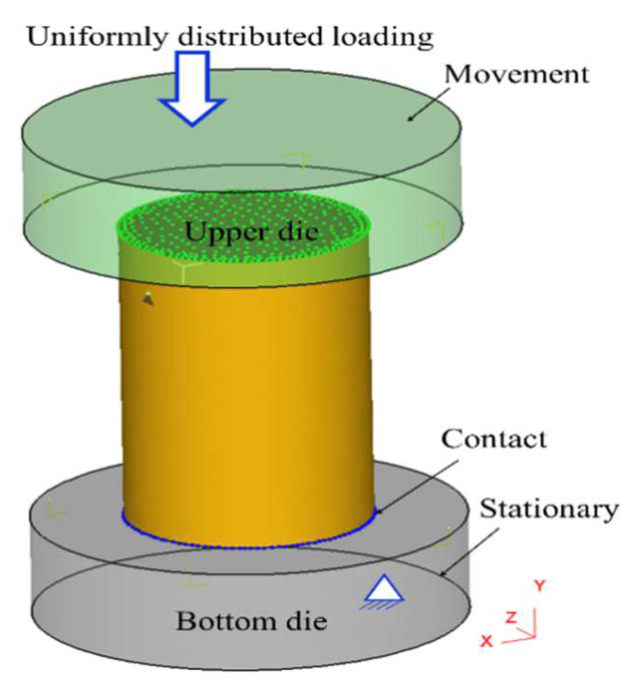
The FEM model for hot compression.

In order to simulate DRX behavior and grain-dimension evolution in this HIPed superalloy during hot compression, the proposed piecewise DRX kinetics equations and grain-growth model are integrated into the DEFORM software through the development of the subprogram. The program flowchart to simulate the DRX behavior and grain size is illustrated in Figure 19. First of all, the hot-compression parameters and the initial microstructure are inputted. Then, for a given time increment, $\varepsilon_{c}$ is calculated. If $\varepsilon <\varepsilon_{c}$, the present operation is continued. At the same time, the current strain rate state ($\dot{\varepsilon }$) is evaluated. If $\varepsilon >\varepsilon_{c}$ and $\dot{\varepsilon }(t)>0$, DRX will occur and the *X*_drx_/grain-size dimension will be counted. If $X_{\mathrm{drx}}>95\%$, full DRX is finished. Meanwhile, the current grain dimension is regarded as the mean DRX grain dimension. If $X_{\mathrm{drx}}<95\%$, the mean grain dimension is calculated according to the grain-growth model. Finally, when the deformation is finished, the *X*_drx_ and grain size are outputted. Figure 20 displays the hot-deformed finite-element geometric model, which is composed of a billet and upper and lower dies. The three-dimensional (3D) transmutable type is applied to the hot-compressed block, whereas the 3D-resolution rigid body is used as a mold. During hot compression, the P/M superalloy workpiece and dies are regarded as the variable object and immutable objects, respectively. The radius of the workpiece is Φ 4 mm and the height is 12 mm. The simulated parameters are the same as those of the present experiments. The moving velocity of the top die is converted from the experimental strain rate. The shear friction is selected and the friction factor is 0.12. The element number of the billet and dies are 30,000 and 8000, respectively.

**Figure 21:**
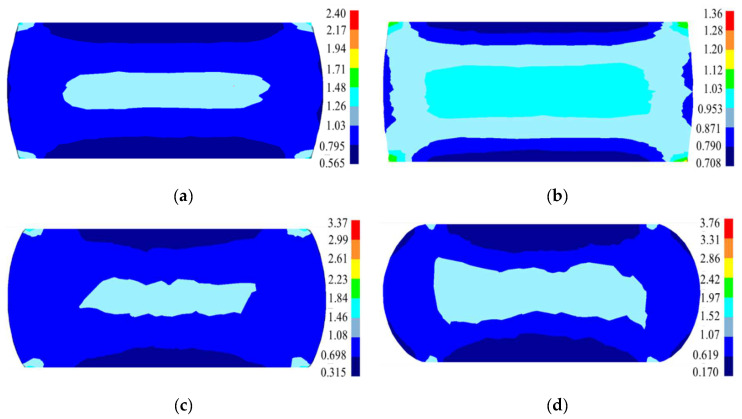
The equivalent plastic strain at (**a**) 1110 °C/0.1 s^−1^; (**b**) 1140 °C/0.1 s^−1^; (**c**) 1110 °C/0.01 s^−1^; and (**d**) 1110 °C/1 s^−1^.

The evolution of the equivalent strain at diverse deformation parameters is demonstrated in Figure 21. The distribution of equivalent strain almost changes symmetrically along the compression axis and radial direction. Three typical deformation regions (large, free, and difficult deformation) are observed. The center is a high-strain area, namely the large deformation zone resulting from the triaxial constringent stress and small frictional force, whereas the end faces that have contact with the dies are low-strain areas, i.e., the difficult deformation regions. Compared with the equivalent strain in the central region, the equivalent strain at the edge and end of the expansion site is smaller. In addition, the uniformity of the strain distribution in the central region is improved with a reduced strain rate or a raised temperature.

**Figure 22:**
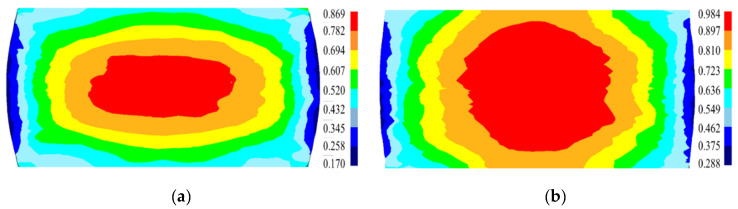

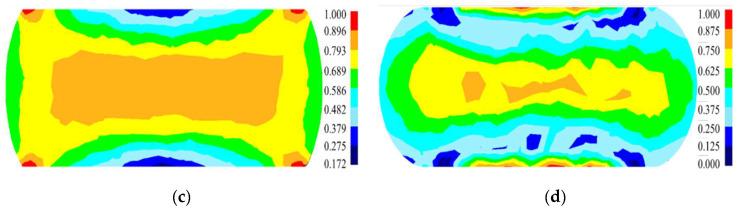
The distribution of the simulated *X*_drx_ at (**a**) 1110 °C/0.1 s^−1^; (**b**) 1140 °C/0.1 s^−1^; (**c**) 1110 °C/0.01 s^−1^; and (**d**) 1110 °C/1 s^−1^.

The distribution of the predicted *X*_drx_ at diverse deformation parameters is displayed in Figure 22. There are obvious differences in the DRX degree in the different regions, which is due to the non-uniformity deformation caused by the friction between the dies and billet. The DRX degree is the highest in the large deformation zone, whereas the lowest is in the difficult deformation region. The simulated *X*_drx_ in the large deformation zone increases with a reduced strain rate or a raised temperature. They are 80%, 97%, 85.9%, and 78.1% at 1110 °C/0.1 s^−1^, 1140 °C/0.1 s^−1^, 1110 °C/0.01 s^−1^, and 1110 °C/1 s^−1^, respectively. These predicted values well agree with the test ones.

**Figure 23:**
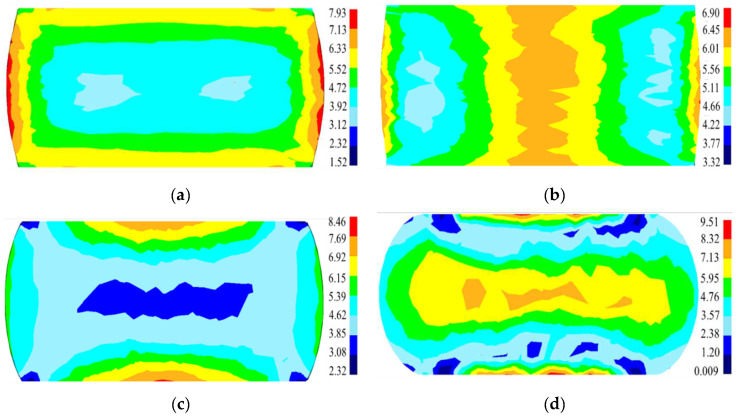
The distribution of the simulated grain size at (**a**) 1110 °C/0.1 s^−1^; (**b**) 1140 °C/0.1 s^−1^; (**c**) 1110 °C/0.01 s^−1^; and (**d**) 1110 °C/1 s^−1^.

**Figure 24:**
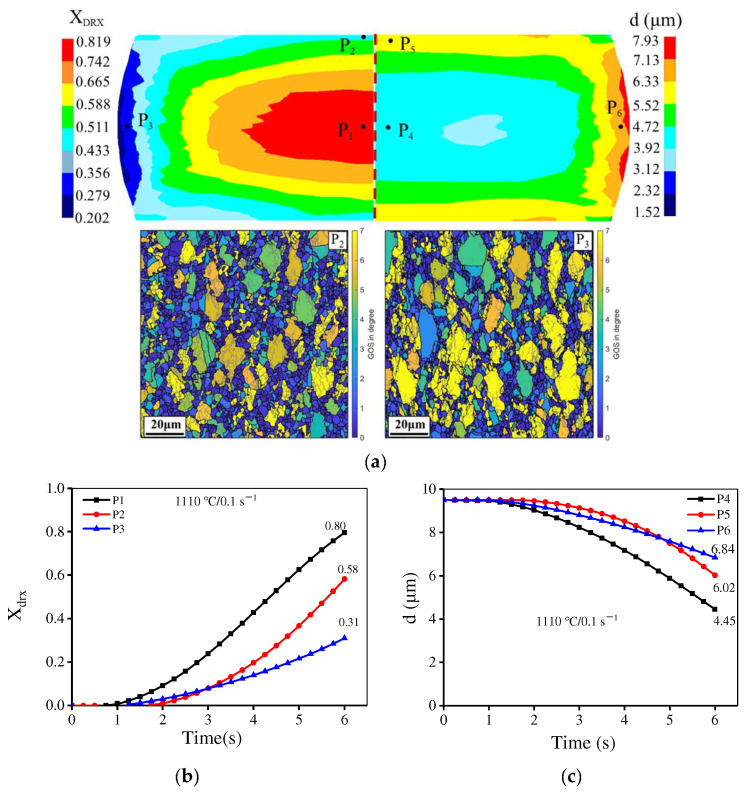
The simulated *X*_drx_ and average grain size in key points at 1110 °C/0.1 s^−1^ (**a**) Distribution of the simulated *X*_drx_ and average grain size; (**b**) The variation of the simulated *X*_drx_ with deformation time; (**c**) The variation of the simulated average grain size with deformation time.

Figure 23 exhibits the distribution of the simulated grain size under different deformation conditions. Obviously, the distribution of grain dimension is non-uniform at disparate deformation parameters. The average grain dimension in the large deformation zone gradually decreases when the strain rate is raised or the temperature is reduced. The experimental average grain sizes in the large deformation zone are 4.21 μm, 7.83 μm, 6.24 μm, and 2.27 μm, respectively, at 1110 °C/0.1 s^−1^, 1140 °C/0.1 s^−1^, 1110 °C/0.01 s^−1,^ and 1110 °C/1 s^−1^, which coincide with the simulated results. The simulated *X*_drx_ and grain sizes in various deformation regions at 1110 °C/0.1 s^−1^ are quantitatively analyzed and are displayed in Figure 24 and Table 3. In Figure 24a, the DRX degree in the large deformation area is high while the average grain dimension is small. Also, the volume fraction of DRX in the non-central position is limited (the points P_2_ and P_3_ in Figure 24a), which is identical to the results from EBSD observation. Figure 24c,d) shows the variations of the simulated *X*_drx_ and average grain size at different positions with deformation time. The comparisons of the experimental/simulated *X*_drx_ and average grain size at 1110 °C/0.1 s^−1^ are shown in Table 3. It can be found that the simulation results well agree with the experimental ones. Thus, the results further indicate that the microstructure evolution can be accurately predicted by the proposed piecewise DRX kinetics equations and grain-growth model.

## 4. Conclusions

The DRX features and grain-growth behavior for a novel P/M superalloy are systematically studied. The piecewise DRX kinetics equations and grain-growth model are developed. The important conclusions are summarized:

1. The DRX behavior and grain features are sensitive to deformation parameters. The raised temperature/true strain can increase the volume fraction of DRX and the mean dimension of DRX grains. As the temperature is reduced or the true strain is raised, the mean grain dimension declines. Increasing the strain rate reduces the DRX volume fraction and mean grain size. Moreover, the main DRX mechanism of the novel P/M superalloy is DDRX.

2. Piecewise DRX kinetics equations are proposed to predict DRX behavior in forming processes. The correlation coefficient of them is 0.992 and the average absolute relative error is 2.3%. Also, an accurate model is established to describe the grain-growth behavior during hot deformation.

3. The piecewise DRX kinetics equations and the grain growth equation are embedded into the DEFORM software using a secondary development method and the DRX features and grain-growth behavior in hot-compression processes are simulated. The simulated results show that the proposed piecewise DRX kinetics equations and grain-growth model can accurately depict the microstructure evolution of this novel P/M superalloy during hot deformation.

## Figures and Tables

**Table 1:** The chemical compositions of the novel P/M superalloy (wt. %).

Al	Ti	Nb	Co	Cr	W	Mo	Ta	Hf	Ni
2.7–3.1	3.6–3.9	1.2–1.3	18–20	11.5–13.5	4.0–4.5	3.5–4.5	0.9–1.2	0.1–0.3	Bal.

**Table 2:** The size of DRX grain (*d*_drx_) at the steady-state deformation stage (μm).

Temperature/Strain Rate	0.001 s^−1^	0.01 s^−1^	0.1 s^−1^	1 s^−1^
1080 °C	3.53	2.23	1.64	1.36
1110 °C	6.98	4.37	3.24	2.32
1140 °C	17.07	12.46	7.52	7.36
1170 °C	33.75	21.73	14.73	10.29

**Table 3:** The comparisons of the experimental/simulated *X*_drx_ and average grain size at 1110 °C/0.1 s^−1^.

Tracked Points	DRX Fraction (%)		Tracked Points	Average Grain Size (μm)	
	Experiment	Simulation		Experiment	Simulation
P_1_	77.4	80	P_4_	4.21	4.45
P_2_	52.8	58	P_5_	5.19	6.02
P_3_	40.8	31	P_6_	6.44	6.84

## Data Availability

The raw/processed data required to reproduce these findings cannot be shared at this time as the data also form part of an ongoing study.
